# Subvesical bile duct and the importance of the critical view of safety: Report of a case

**DOI:** 10.1016/j.ijscr.2019.05.040

**Published:** 2019-05-28

**Authors:** Constantine P. Spanos, Marianna P. Spanos

**Affiliations:** Aristotelian University, Fitziou 15, N751, Panorama-Thessaloniki, 55236, Greece

**Keywords:** Laparoscopic, Cholecystectomy, Subvesical duct, Case report

## Abstract

**Introduction:**

This is a case report in which a subvesical duct was identified intra-operatively during laparoscopic cholecystectomy.

**Presentation of case:**

A 49-year-old man underwent elective laparoscopic cholecystectomy for symptomatic cholelithiasis. During the procedure, the cystic duct and cystic artery were dissected. After ligation and division of these structures, a subvesical bile duct draining directly into the gallbladder was noted. In the present case, the subvesical duct was ligated and divided. The patient had an uneventful recovery.

**Discussion:**

Subvesical bile ducts, also known as ducts of Luschka, represent anatomic variations of the biliary tree in which one or more bile ducts traverse in close contact with the gallbladder fossa. Inadvertent and undetected injury of these ducts are a frequent cause of cholecystectomy-associated bile leaks.

**Conclusion:**

Application of the critical view of safety principle may assist in intra-operative detection of aberrant biliary anatomy, thus preventing iatrogenic injury.

## Highlights

• The critical view of safety is an important principle in performing laparoscopic cholecystectomy.
• Aberrant anatomy is a major cause of biliary injury during laparoscopic cholecystectomy.
• Using the critical view of safety principle may prevent such injuries.

## 1 Introduction

Laparoscopic cholecystectomy is a common procedure in general surgery. The main challenges a surgeon may confront include inflammation, ductal and/or vascular injury, and aberrant anatomy. We present a case in which a subvesical bile duct was found during laparoscopic cholecystectomy.

This work has been reported in line with the SCARE criteria [7].

## 2 Report of a case

**Fig. 1:**
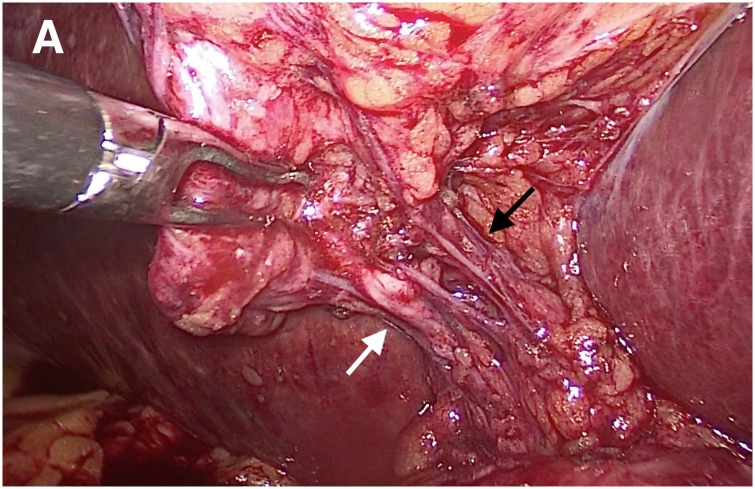
The cystic duct (white arrow) and cystic artery (black arrow) have been dissected with the critical view of safety principle.

**Fig. 2:**
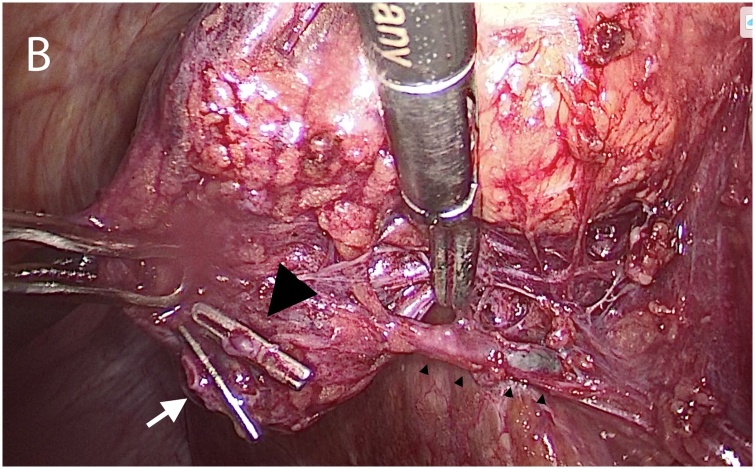
The cystic duct (white arrow) and cystic artery (large arrowhead) have been divided. A subvesical duct connecting the gallbladder infundibulum to the right hepatic lobe is detected (small arrowheads). Histopathology confirmed this to be a bile duct.

A 59-year-old man underwent laparoscopic cholecystectomy for symptomatic cholelithiasis. Carboperitoneum was established using a standard open Hasson technique. Four laparoscopic ports were placed in standard fashion [8]. During the procedure, the cystic duct and artery were dissected using the critical view of safety principle. (Fig. 1) After ligation and division of these structures, the gallbladder was carefully dissected with monopolar diathermy from its fossa in the liver. A subvesical bile duct was detected, originating from the right hepatic lobe/gallbladder fossa, draining into the gallbladder infundibulum (Fig. 2). The duct was carefully dissected, controlled with clips, and divided. The patient had an uneventful recovery. Histopathological examination of the resected specimen confirmed the existence of the subvesical duct.

## 3 Discussion

Gallstone disease remains amongst the most common surgical diseases in Western societies and minimally invasive cholecystectomy is one of the most common surgical procedures performed. A fundamental tenet of surgical anatomy is the structural variability of the biliary tree. A common anatomic variation with surgical implications is the subvesical bile duct. This has been termed, possibly incorrectly, as the “duct of Luschka” [1]. In his anatomic textbook, Herbert von Luschka described intra-mural glands draining into the gallbladder lumen, as well as a network of microscopic ducts within the soft tissue surrounding the gallbladder, and not subvesical bile ducts [1,2].

The clinical importance of the subvesical duct lies with the potential risk for injury during cholecystectomy and hepatobiliary procedures. Bile leaks occur in approximately 0.2–2% of minimally invasive cholecystectomies [3]. Inadvertent and undetected subvesical duct injury is a frequent cause of bile leaks during cholecystectomy; up to 27% of leaks have been attributed to such injuries [1,3].

Schnelldorfer et al. have attempted to categorize subvesical ducts into [1] segmental or sectorial subvesical bile ducts [2], accessory subvesical bile ducts [3], hepaticocholecystic bile ducts, and [4] aberrant subvesical bile ducts [1]. In our case, we possibly encountered a right lobar hepaticocholecystic bile duct.

The critical view of safety principle has been utilized to identify the cystic duct and artery during laparoscopic cholecystectomy. This method will prevent misidentification of the common bile duct or aberrant ducts as the cystic duct. Significant bile duct injury will thus be avoided [5]. In order to achieve a critical view of safety three conditions must be met: firstly; the hepatocystic triangle must be cleared of fat and fibrous tissue, second; the lower third of the gallbladder must be separated from the liver to expose the cystic plate, and finally; two, and only two structures must be seen entering the gallbladder [5]. In our case, the subvesical bile duct was identified after division of the cystic duct and artery. A more thorough dissection of the lower third of the gallbladder from the liver may have revealed the subvesical duct prior to division of the aforementioned structures.

An intraoperative cholangiogram was not obtained during our case. Relative indications for intraoperative cholangiography during laparoscopic cholecystectomy include a history of jaundice, pancreatitis, hyperbilirubinemia or a dilated common bile duct on preoperative ultrasonography [6]. These were absent in our patient.

Subvesical bile ducts can be depicted preoperatively with specialized imaging techniques [3]. However, standard preoperative imaging (namely, ultrasonography) will not detect these ducts in the vast majority of cases. Careful dissection during cholecystectomy utilizing the critical view of safety principle [4], as well as high definition laparoscopy, may allow for intraoperative detection, dissection and control of subvesical ducts. The use of three-dimensional (3D) laparoscopy may achieve better depth-perception on the surgeon’s part, thus facilitating identification of normal and aberrant biliary anatomy [9]. Fluorescent intraoperative cholangiography with indocyanine green (ICG) was first described by Ishizawa et al. [10]. Its use has led to identification of aberrant biliary ducts during laparoscopic cholecystectomy [11]. The importance of intraoperative bile leak identification should also be emphasized. [12]. Currently available advanced technology described above can be utilized to identify and prevent major cholecystectomy-associated bile leaks.

## Conflict of interest

There are no conflicts of interest regarding this article.

## Funding

There are no sources of funding regarding this article.

## Ethical approval

On the basis of this being a case report, the Institutional Review Board of the Aristotelian University does not mandate that ethical approval is required. Thus, this case report is exempt from the Institutional Review Board Approval process.

## Consent

Appropriate consent regarding this article has been granted by the patient.

## Author contribution

Constantine Spanos: concept, design, final proofreading.

Marianna Spanos: manuscript preparation, image preparation.

## Registration of research studies

N/A.

## Guarantor

Guarantor: Constantine P. Spanos, MD.

## Provenance and peer review

Not commissioned, externally peer-reviewed.
